# Predictors of COVID-19 vaccination intention among Iranian population: applying the theory of planned behavior

**DOI:** 10.3205/dgkh000424

**Published:** 2022-11-08

**Authors:** Majid Barati, Saeed Bashirian, Jalal Abdi, Mohadeseh Sadri, Maryam Afshari, Malihe Taheri, Salman Khazaei, Masomeh Rostami-Moez

**Affiliations:** 1 Department of Public Health, School of Health, Social Determinants of Health Research Center, Hamadan University of Medical Sciences, Hamadan, Iran; 2 Public Health, Health Center of Hamadan, Hamadan University of Medical Sciences, Hamadan, Iran; 3 MSc of Health Education, Department of Public Health, School of Health, Hamadan University of Medical Sciences, Hamadan, Iran; 4 Department of Public Health, School of Health, Hamadan University of Medical Sciences, Hamadan, Iran; 5 Social Determinants of Health Research Center, Hamadan University of Medical Sciences, Hamadan, Iran; 6 Research Center for Health sciences, Hamadan University of Medical Sciences, Hamadan, Iran; 7 Research Center for Health Sciences, Hamadan university of Medical Sciences, Hamadan, Iran

**Keywords:** intention, vaccination, COVID-19, theory of Planned Behavior, Iran

## Abstract

**Background:**

The present study was conducted to assess the determinants of the intention to receive the COVID-19 vaccination among the Iranian population.

**Methods:**

In this cross-sectional study, 1,056 individuals of the general population living in Hamadan County were studied from April to June 2021. Using a proportional stratified sampling method, subjects were selected from those covered by 34 comprehensive health service centers and health bases. Then, the subjects were selected by simple random sampling. Data were collected by sending a questionnaire link to eligible individuals, who completed it online. The questionnaire determined demographic data and constructs of the Theory of Planned Behavior.

**Results:**

1,056 subjects answered the study; women accounted for 57.9% of the respondents. According to the findings, the majority of respondents (79.5%) reported that they would strongly agree and agree to get vaccinated against COVID-19. Older age, male gender, occupation, underlying chronic disease, death of relatives and friends due to COVID-19, and history of influenza vaccinations were significantly related to the intention to get vaccinated against COVID-19 (P>0.05). Also, the intention was associated with increased scores in the attitude toward the behavior, subjective norm, and perceived behavioral control.

**Conclusion:**

Despite doubt about the details of the intention to receive COVID-19 vaccination, most subjects reported intending to be vaccinated against COVID-19, but the real rate of vaccination may be lower. Vaccination intention reflects overall vaccination attitudes, subjective norms, and perceived behavioral control.

## Zusammenfassung

**Zielsetzung:**

Die Studie wurde durchgeführt, um die Determinanten für die Absicht, sich mit COVID-19-Impfstoff impfen zu lassen, in der iranischen Bevölkerung zu ermitteln.

**Methode:**

In die Querschnittsstudie wurden 1.056 Personen der Allgemeinbevölkerung im Bezirk Hamadan, Iran, von April bis Juni 2021 eingeschlossen. Mithilfe einer proportionalen, geschichteten Stichprobenmethode wurden die Probanden aus 34 Gesundheitszentren bzw. Gesundheitsstützpunkten ausgewählt. An die mittels einfacher Zufallsstichprobe ausgewählten Probanden wurde ein Fragebogen-Link versandt und online ausgefüllt. Mit dem Fragebogen wurden demografische Daten und Konstrukte der Theorie des geplanten Verhaltens ermittelt.

**Ergebnisse:**

1.056 Personen nahmen an der Studie teil; davon waren 57,9% Frauen. 79,5% der Teilnehmer gaben an, dass sie der Impfung gegen COVID-19 ohne Einschränkung zustimmen. Höheres Alter, männliches Geschlecht, Beruf, chronische Grunderkrankung, Tod von Verwandten und Freunden durch COVID-19 und frühere Grippeschutzimpfung waren signifikant mit der Impfbereitschaft assoziiert (p<0,05). Außerdem war die Absicht zur Impfung mit einem Anstieg der Werte für die Einstellung zum Verhalten, der subjektiven Norm und dem wahrgenommene Verhaltensscore verbunden.

**Schlussfolgerung:**

Ungeachtet des Zweifels an einzelnen Gründen für die Bereitschaft zur COVID-19-Impfung gaben die meisten Probanden an, sich gegen COVID-19 impfen lassen zu wollen; die tatsächliche Inanspruchnahme ist jedoch möglicherweise geringer. Die Impfabsicht spiegelt die allgemeine Einstellung zum Impfstoff, subjektive Normen und die wahrgenommene Verhaltenskontrolle wider.

## Introduction

Coronaviruses are a large family of viruses that can cause diseases ranging from the common cold to more serious illnesses such as MERS & SARS. The latest type of the virus is the acute respiratory syndrome coronavirus, which causes COVID-19 disease [1]. Symptoms of COVID-19 disease can range from the common cold to fever and chills, cough, shortness of breath, acute respiratory problems, and gastrointestinal problems [2]. In addition to the respiratory system, coronavirus can affect other vital organs in the body, including kidneys, heart, and brain. The virus can also increase the risk of death and death in the elderly and people with high blood pressure, heart disease and diabetes [3]. 

To prevent the spread of the coronavirus, all governments have introduced restrictions such as social distancing plans, cancellation of gatherings, travel restrictions, closure of schools, universities and shopping malls, home quarantine, public education, screening and vaccination [4]. Currently, one of the main and most important global strategies to control the coronavirus epidemic is to vaccinate the population of countries. Vaccination is the most effective way to control infectious diseases, especially among high-risk groups. In addition to providing health and reducing morbidity and mortality, vaccination reduces the cost of treatment, the use of drugs and economic problems [5]. Immunization programs are successful only when there is a high level of acceptance and coverage in these programs [6]. To achieve this, it is vital to accept the COVID-19 vaccination and to trust the vaccination based on the intent to vaccinate people against COVID-19 [7]. 

In a general definition, Ajzen [8] defines intention as a person’s readiness to perform a particular behavior and refers to a person’s decision to perform a behavior. It is assumed that intention can represent the efforts that a person may make to achieve a goal or correct behavior. One of the methods to study human behavior and its predictors is the use of socio-psychological models/theories. Therefore, to achieve the effective factors of vaccination intention, the theory of planned behavior (TPB) is used.

**Figure 1:**
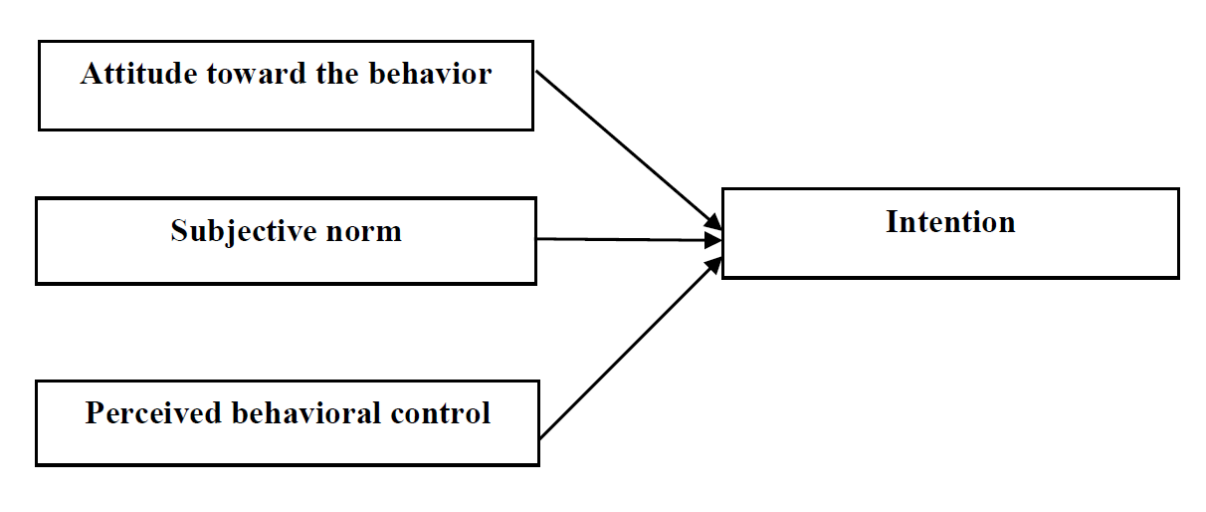
Schematic representation of the theory of planned behavior (TPB) constructs

The TPB is one of the theories that has been widely used to explain the intention to do work. In this theory, intention is understood as a function of three important predictors: attitude, social or mental norms, and perceived behavioral control (Figure 1 (Fig. 1)). Attitude is the favorable or unfavorable evaluation (positive or negative) of a behavior, or in other words, the general evaluation of the advantages and disadvantages of performing a particular behavior. In addition, perceived social pressures to perform or not to perform a behavior are defined by social norms, and the ease or difficulty of performing a behavior is defined as perceived behavioral control. Social norms refer to people’s perceptions of opposition or approval of other important people towards performing a behavior. Perceived behavioral control also refers to people’s belief that they can perform a particular behavior [8], [9]. 

At present, little is known about the determinants of vaccination intent among Iranians at the height of the COVID-19 epidemic. According to the research team, most research is related to the prevalence, epidemiological identification and clinical features of infected patients [10], [11], virus genomic characteristics [12], global health challenges [13] and incorrect information in this field [14]. Therefore, the present study was conducted to assess the determinants of the intention to receive the COVID-19 vaccination among the general population

## Materials and methods

### Settings

In this cross-sectional study, 1,056 individuals of the general population living in Hamadan County in western Iran who were confronted with COVID-19 were included from April to June 2021. 

### Study sample

We used multistage sampling for choosing participants. At first, through stratified sampling, subjects were selected from 34 comprehensive health service centers and health posts in Hamadan County, proportional to their size. Then, based on dedicated samples from each facility, the subjects were selected from comprehensive health service centers and health posts by a simple random sampling. We cautiously chose our subjects from lists of the integrated health system in these facilities. Based on the results obtained from a previous study [15], which showed that 25% of people did not intend to be vaccinated against COVID-19 (p), a margin of error=0.025 (d), and a confidence interval of 95%, the required sample size was calculated to be 1,152. 

Inclusion criteria for participation in the study were being over 18 years old and literate. Exclusion criteria were refusal to cooperate in the research and not having an Android or iPhone phone. 

### Data collection

Data were collected by sending a questionnaire link to eligible individuals, who completed it online. An author-developed questionnaire consisted of two parts to document:

1. demographic information including age, sex, education level, marital status, occupation, income, underlying chronic disease, history of coronavirus disease, death of relatives and friends due to COVID-19, and history of influenza vaccination;
2. data relating to constructs of the TPB.

The intention, attitude toward the behavior, subjective norm, and perceived behavioral control were assessed by a Likert scale: strongly agree (5 points), agree (4 points), no idea (3 points), disagree (2 points), and strongly disagree (1 point). Thus, the variety of potential points for the construct of intention was 2 to 10 (e.g., I’m going to get the COVID-19 vaccination), the construct of attitude toward the behavior was 8 to 40 (e.g., the COVID-19 vaccination reduces the severity of the disease), the subjective norm was 8 to 40 (e.g., healthcare professionals advise me to get the COVID-19 vaccination), perceived behavioral control was 5 to 25 (e.g., despite worrying about the side effects of the vaccine, I will agree to be vaccinated). 

### Pilot testing of the questionnaire

To assess the content validity, the questionnaire was distributed among 10 health education and health promotion specialists, and was finally approved by the experts by estimating the values of content validity ratio (CVR) and content validity index (CVI) for questions and making necessary changes. The reliability of the questionnaire was assessed using the internal consistency method among a group of 30 people in the target population. Cronbach’s alpha of attitude was estimated to be 0.78, 0.86 for subjective norms, 0.77 for perceived behavioral control and 0.93 for intention.

### Ethical approval

First, the objectives of the study were explained to the participants by telephone, and after obtaining informed consent from the participants, a questionnaire link was sent to them. This study was accepted by the Ethics Committee of Hamadan University of Medical Sciences (IR.UMSHA.REC.1400.151). 

### Data analysis

Data analysis was performed using SPSS24. The significance level for the tests was <5%. Test statistics including ANOVA, Pearson correlation coefficient, independent t-tests, and linear regression analysis were used to describe the association between variables and constructs. 

## Results

1,056 individuals participated in this study; thus, the response rate was 91.6%. The 96 subjects who were excluded from the study did not consent to answer the questionnaire. The mean ±SD of the age of the study participants was 40.71±12.41 years. 

As shown in Table 1 (Tab. 1), the majority of participants were female (57.9%), most participants were in the age group 31–40 years (37.2%), and their highest level of education was college (50.4%). Also, the majority of participants were married (85%). 37.3% of the participants listed their occupation as “housewife”, and most had a moderate income (62.8%). The majority of participants did not have an underlying chronic disease (81.9%). About 29% of the participants reported a history of coronavirus disease in themselves. In addition, 33.4% of the samples reported the death of relatives and friends due to COVID-19. About 71.1% of the participants did not report a history of influenza vaccination. 

The results showed a positive correlation (r=0.539, P<0.01) between attitude toward the behavior and subjective norm to receiving the COVID-19 vaccination. Moreover, attitude to being vaccinated against COVID-19 was positively correlated with perceived behavioral control (r=0.575, P<0.01) and intention (r=0.648, P<0.01). Furthermore, the subjective norm towards getting vaccinated against COVID-19 was positively correlated (r=0.583, P<0.01) with perceived behavioral control and intention (r=0.651, P<0.01). There was a positive correlation between perceived behavioral control and intention (r=0.650, P<0.01) to receiving the vaccination against COVID-19 (Table 2 (Tab. 2)).

79.5% answered that they strongly agreed and agreed to be vaccinated against COVID-19 and 73.4% strongly agreed and agreed that they intended to register in the vaccination system. 

The results showed that attitude to receiving the COVID-19 vaccination had a significant relationship with education level, age, sex, occupation, income, death of relatives and friends due to COVID-19, and history of influenza vaccination (p<0.05). In addition, subjective norm was significantly related to occupation, income, underlying chronic disease, death of relatives and friends due to COVID-19, and history of influenza vaccination (P<0.05). Also, there was a significant relationship between perceived behavioral control and age, education level, occupation, income, underlying chronic disease, death of relatives and friends due to COVID-19, history of influenza vaccination (P<0.05). However, the other constructs of the theory of planned behavior were not statistically significantly related to the demographic and background variables. As presented in Table 3 (Tab. 3), intention was significantly related to sex, age, occupation, underlying chronic disease, death of relatives and friends due to COVID-19, and history of influenza vaccination (p<0.05). 

As shown in Table 4 (Tab. 4), one unit of increase in the attitude toward the behavior and subjective norm score was associated with the mean score intention increasing by 0.31. In addition, with one unit of increase in the score of perceived behavioral control, the mean score of intention increased by 0.28.

## Discussion

This is the first study to comprehensively describe predictors of factors influencing in intention to receive the COVID-19 vaccination Iran.

During the initial months of the COVID-19 pandemic, we awaited the development and availability of safe and effective COVID-19 vaccines. Nevertheless, the success of any vaccination plan depends on high vaccination rates [15]. Similar to the findings of previous studies [16], [17], our study indicated that high proportions of the Iranian public intended to be vaccinated COVID-19 once a vaccine had become available, so that an effective vaccine would end the pandemic. However, in contrast to previous research [18], COVID-19 vaccination intentions were weak among adults. One reason for this may be associated with inadequate factors affecting the individual’s intention to receive the vaccination.

In addition, our results recognize numerous factors underlying the reasons why some persons are more ready to be vaccinated against COVID-19. We found that attitude toward the behavior, subjective norms and perceived behavioral control about COVID-19 vaccination explained 59% of the variance in vaccination intention. These findings show the importance of people’s beliefs, such as attitudes, subjective norm, and perceived behavioral control in accepting why some individuals engage in a behavior whereas others do not, which supports the hypotheses of the TPB [8]. 

Similar to another study [19], significantly, we found that the construct that clarified the greatest percentage of the variance in intention to be vaccinated against COVID-19 was the attitude toward the behavior. Thus, the results indicate that individuals may hold certain attitudes toward the vaccination and this overall attitude controls the intention to receive the COVID-19 vaccination at this point. In addition, these results indicated that the positive attitude to prevention of COVID-19 may contribute to a higher vaccination acceptance.

Based on TPB constructs, subjective norms can determine health behaviors, such as getting vaccinated against COVID-19. This result can be influenced by the positive attitude of other important people to encourage the individual to get the COVID-19 vaccination. Similar to the findings of a previous study [20], the comments of other people important to the individual leads to the likelihood of intention to receive the COVID-19 vaccination. To promote COVID-19 vaccination intention, it is important to strengthen the subjective norms that this preventive measure is absolutely necessary to protect other members of the community [21]. However, in contrast to previous research [22], the subjective norm was not a significant predictor for the intention to accept COVID-19 vaccination in mainland Chinese university students. This is probably because the target groups of the two studies are different.

Based on our findings, we found that vaccination intention was related to greater perceived behavioral control about COVID-19 vaccination. Other studies support this finding [22], [23]. Perceived behavioral control was not a predictor for the intention to be vaccinated against COVID-19. It seems that the difference in the reported results is due to differences in the type of communities studied. 

Consistent with other studies [19], we found that seasonal influenza vaccination was strongly related to the intention to receive the COVID-19 vaccination. These people have a history of not becoming ill with influenza thanks to the vaccination, so they showed a greater intention to receive the COVID-19 vaccination. In addition, we found that older age and male gender were related with a greater intention to be vaccinated. Other studies support this finding [15], [19]. It may reflect the well-publicized increased risk of disease and death among older people and males due to COVID-19 [24]. In this survey, we observed that chronic medical conditions and the death of relatives and friends due to COVID-19 were associated with a greater intention to be vaccinated. This result was consistant with the results of a previous study [15]. It seems that these people weree more aware of the risk of the disease, and vaccination is especially important to them. Supporting this (19), our study found that people with a better occupational status were more likely to be vaccinated against COVID-19. It is likely that better work conditions increase the perceived risk of COVID-19 to oneself and thus intention to be vaccinated.

The findings of this research should be understood in light of limitations. First, the study did not evaluate some psychological factors that may be associated with vaccination intention. Also, the getting of COVID-19 vaccine changes over time.

## Conclusions

Despite doubt about the details of the intention to receive the COVID-19 vaccination, most subjects reported intending to be vaccinated against COVID-19, but the real rate of vaccination may be lower. Vaccination intention reflects overall vaccination attitudes, subjective norms, and perceived behavioral control. Targeted interventions such as health education programs are needed in the future to increase the intention to receive the COVID-19 vaccination.

### Highlights

- The majority of participants strongly agreed and agreed to get vaccinated against COVID-19
- The intention was associated with an increase in scores for attitude toward the behavior, subjective norm, and perceived behavioral control.

## Notes

### Competing interests

The authors declare that they have no competing interests.

### Acknowledgments

This project was approved by the Research and Technology Deputy of Hamadan University of Medical Sciences.

### Funding

This work was supported by Hamadan University of Medical Sciences.

## Figures and Tables

**Table 1:**
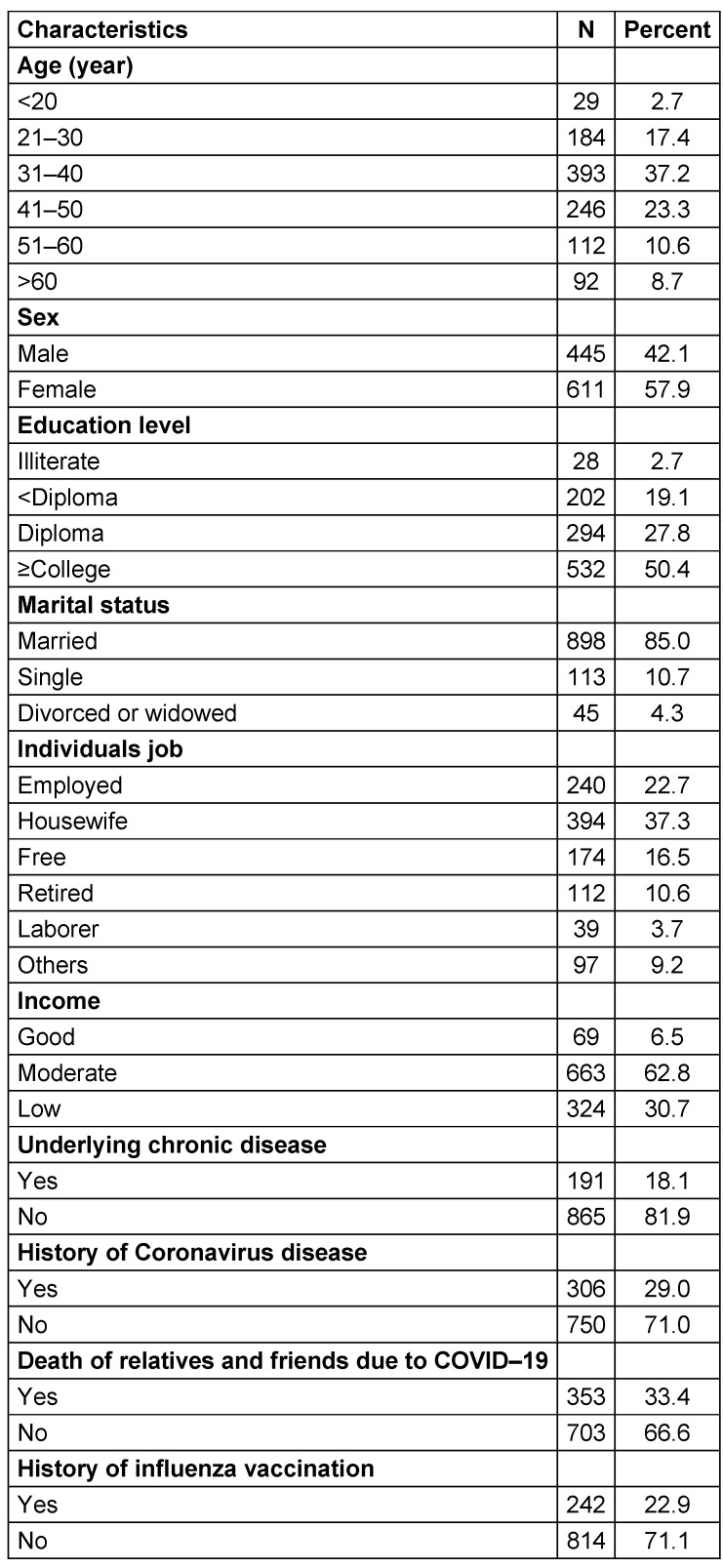
Demographic and background characteristics of the participants (n=1056)

**Table 2:**
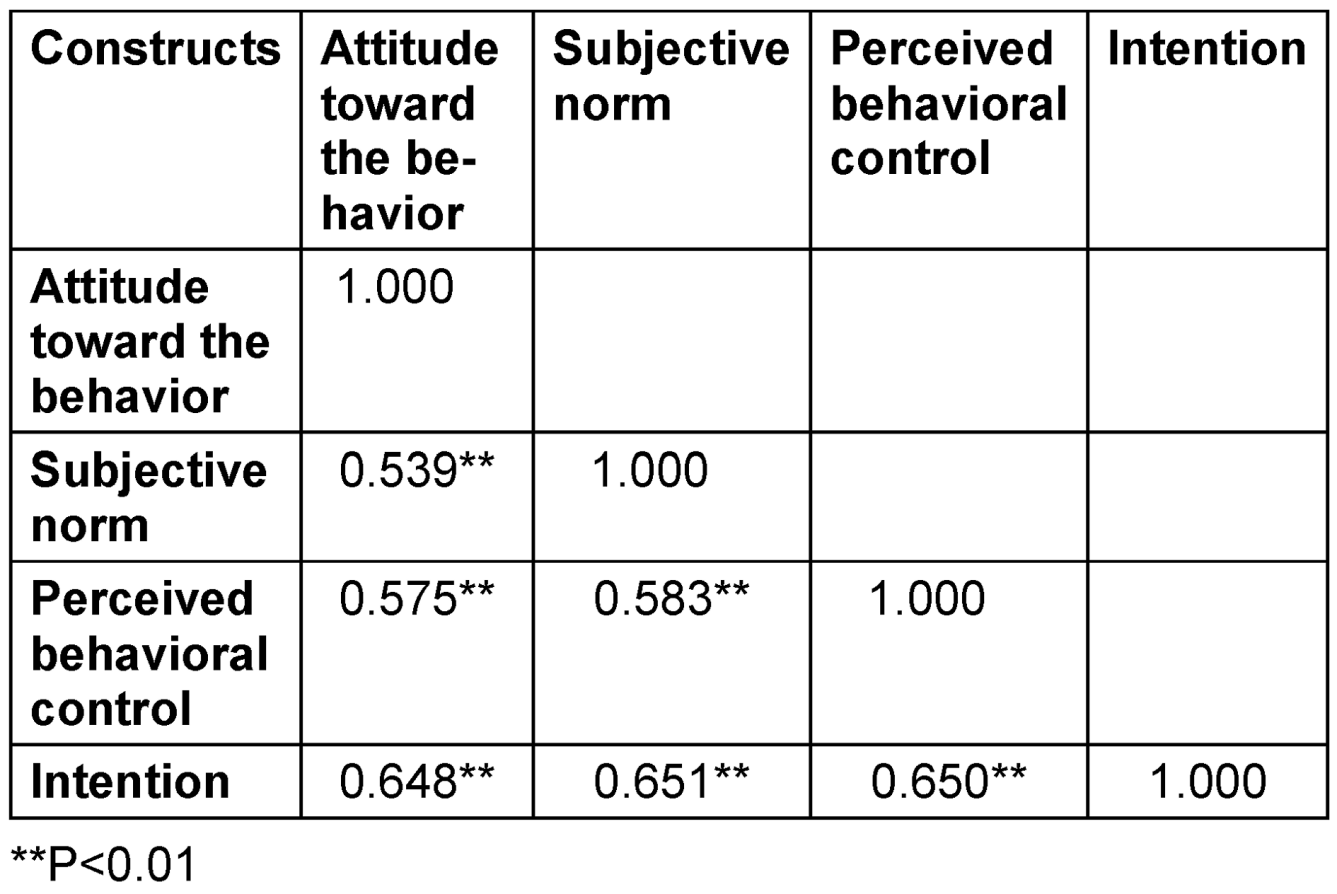
The correlation coefficient matrix the theory of planned behavior (TPB) (n=1056)

**Table 3:**
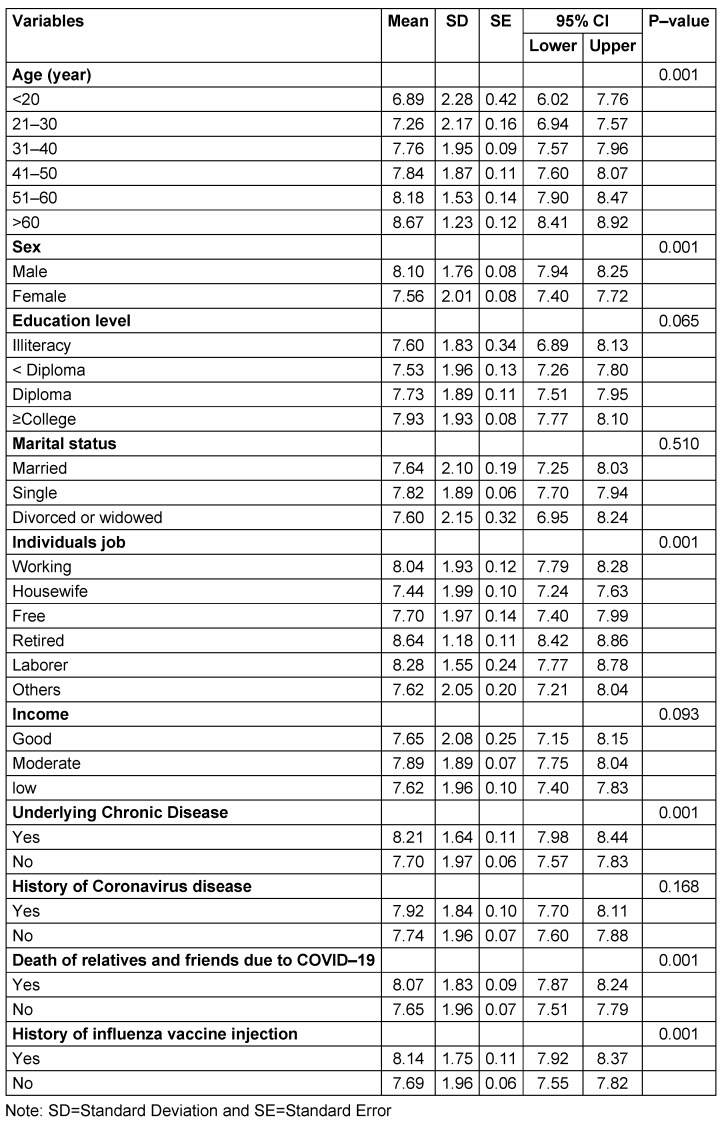
The relationship of intention with demographic and background characteristics (n=1056)

**Table 4:**
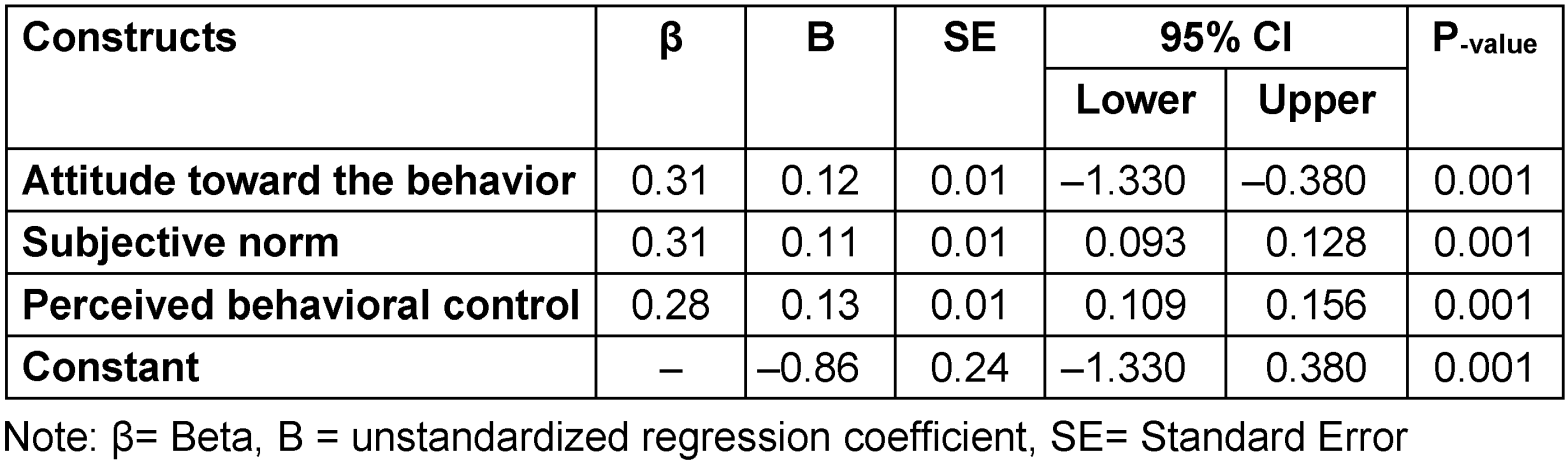
Predicting intention of COVID-19 vaccine injection among participants: Linear regression analyses (n=1056; adjusted R^2^=0.593)
